# Effect of Nitrogen on Phosphine-Susceptible and -Resistant Populations of Stored Product Insects

**DOI:** 10.3390/insects11120885

**Published:** 2020-12-15

**Authors:** Maria K. Sakka, Fotini Gatzali, Vaios T. Karathanos, Christos G. Athanassiou

**Affiliations:** 1 Laboratory of Entomology and Agricultural Zoology, Department of Agriculture, Crop Production and Rural Environment, University of Thessaly, 38446 Nea Ionia, Magnesia, Greece; athanassiou@agr.uth.gr; 2 Agricultural Cooperatives’ Union of Aeghion, 25100 Aeghion, Greece; fgatzali@pesunion.gr (F.G.); vkarath@hua.gr (V.T.K.); 3 Department of Nutrition, Harokopion University, 17671 Athens, Greece

**Keywords:** nitrogen, phosphine resistance, stored product insects, controlled atmospheres

## Abstract

**Simple Summary:**

The sawtoothed grain beetle, the red flour beetle and the rice weevil are three major stored product insects that attack different stored products worldwide. Limited chemical options are available, and eco-friendlier management strategies are needed. Low oxygen treatment can be used as an alternative method to limit chemical treatments. Therefore, we conducted nine trials in commercial nitrogen chambers with phosphine susceptible and resistant populations. The vials with insects were placed in different locations inside the chambers and mortality was recorded after the termination of each trial. The vials were kept at laboratory conditions for 65 days in order to measure progeny production. Low or no survival was recorded in all cases for all species. Moreover, progeny production was suppressed with some exceptions for some species and populations. The current study indicates that low oxygen is effective against phosphine susceptible and resistant populations and can be used as alternative method to chemicals.

**Abstract:**

In this study, we evaluated nitrogen treatment on phosphine-resistant field and -susceptible laboratory populations of different stored product beetles. Nine trials were conducted in commercial nitrogen chambers with the O_2_ level set at 1.0%. Two different temperatures—i.e., 28 and 40 °C—and three exposure intervals—i.e., 2.5, 3 and 9 d—were used in our tests. Adults of the sawtoothed grain beetle, *Oryzaephilus surinamensis* (L.) (Coleoptera: Silvanidae); the red flour beetle, *Tribolium castaneum* (Herbst) (Coleoptera: Tenebrionidae); and the rice weevil, *Sitophilus oryzae* (L.) (Coleoptera: Curculionidae) were used in the trials. The insects were placed in vials with different commodities per species and population, and their mortality was measured after the termination of each trial. Then, the vials were kept in incubator chambers at 25 °C and 65% relative humidity for 65 d to measure progeny production. Complete parental mortality was observed in all cases for *O. surinamensis* and *S. oryzae*, but there was some survival for *T. castaneum* at 28 °C and 3 d of exposure. In general, progeny production was completely (100%) suppressed, with some exceptions for all species and populations. The results indicate that low oxygen is effective for all species tested, regardless of their resistance status to phosphine, and can be further adopted as an alternative method to mitigate resistance in stored product beetles.

## 1. Introduction

Phosphine, the most common fumigant in stored product protection, despite its proved efficacy in a wide range of application scenarios, is now facing its bigger challenge: the development of resistance by many stored product insect populations in several parts of the world [1]. Major stored product insects such as the lesser grain borer, *Rhyzopertha dominica* (F.) (Coleoptera: Bostryhidae); the rusty grain beetle, *Cryptolestes ferrugineus* (Stephens) (Coleoptera: Laemophloeidae); and the red flour beetle, *Tribolium castaneum* (Herbst) (Coleoptera: Tenebrionidae) were found to be resistant to phosphine [1,2,3,4]. The increase in resistance populations worldwide necessitates the need for the use of alternative methods, either as standalone applications or as “resistance breakers” under a rotation concept with phosphine [1,4,5,6,7,8]. Controlled atmosphere has been used for control of insects in a number of fresh commodities—e.g., fruits [9].

There are certain methods that have been evaluated as alternatives to phosphine [7]. For example, the utilization of contact insecticides has been proven to be effective for the control of populations that are resistant to phosphine, while several studies document the absence of cross resistance [10,11]. Recently, Agrafioti et al. [8] reported that heat treatment could be a viable method for the control of phosphine-resistant insect populations. Additional methods that have been proved effective for this purpose in laboratory and field trials are ozone [12] and the fumigant sulfuryl fluoride [13]. All of the above can be used in a phosphine resistance mitigation strategy in different types of facilities and commodities. However, phosphine substitution by some of these methods may not always be feasible, due to the different characteristics of each application scenario. For instance, heat is usually applied in empty spaces in food and processing facilities, and not directly on the commodity, which leaves a certain application gap there in terms of phosphine replacement. Similarly, contact insecticides can substitute for phosphine on raw grains where they can be applied directly as grain protectants, but cannot be used in processed commodities, such as bags of flour or pallets of pasta.

Controlled atmospheres are based on low oxygen environment which kills insects, through oxygen (O_2_) replacement with another gas. Elevated carbon dioxide (CO_2_) levels cause spiracles to open resulting in insect death from anoxia/hypoxia. Controlled atmospheres by using carbon dioxide has direct toxic effects on the insect nervous system, and causes acidification in the hemolymph and failure of membrane in insect tissues [14]. Ofuya and Reichmuth [15] emphasized the importance and the wide applicability of modified atmospheres as environmentally friendly and economically feasible methods that can be further utilized at the post-harvest stages of agricultural commodities. In most cases, reduction in the oxygen level is achieved through the increase in the levels of either nitrogen or carbon dioxide [16], with nitrogen becoming more popular during recent years [14,17,18]. Nitrogen is approximately 78% in the atmosphere and can be easily elevated in a target facility through nitrogen generators or pumps that are able to take the nitrogen from the air and introduce the gas in the area that is to be treated [19,20]. In such an application, the oxygen level is usually kept at 1% or less [21]. Athanassiou et al. [18] tested nitrogen in commercial chambers and found that the increase in the temperature during the application from 28 to 38–43 °C increased the efficacy of the method and sufficiently shortened its application period. In that study, the authors found that the use of nitrogen under increased temperatures could successfully control different life stages of the confused flour beetle, *Tribolium confusum* Jacquelin du Val (Coleoptera: Tenebrionidae); the tobacco moth, *Ephestia elutella* (Hübner) (Lepidoptera: Pyralidae); and *Oryzaephilus surinamensis* (L.) (Coleoptera: Silvanidae) [18]. The use of atmospheric nitrogen can be implemented in different types of facilities, ranging from chambers to large silos [14,15,16].

Considering the above and taking into account the wide applicability of nitrogen in stored product protection, there are no data available for the utilization of nitrogen for the control of insects that are resistant to phosphine. Although nitrogen and phosphine have different modes of action, both act through the insect respiration system, so it is unclear if there are certain interactions that may affect the efficacy of this method. It is now well established that, for some species, phosphine resistant individuals have modified their respiration parameters, as compared with the susceptible individuals [22]. Therefore, the objective of the present study was to evaluate the effect of nitrogen treatment on insect populations with different susceptibility to phosphine. For this purpose, one internal grain feeder, *Sitophilus oryzae* (L.) (Coleoptera: Curculionidae), and two external grain-feeders, *T. castaneum* and *O. surinamensis*, were selected.

## 2. Materials and Methods

### 2.1. Test Insects

The test insects used were reared at the Laboratory of Entomology and Agricultural Zoology (LEAZ), Department of Agriculture, Crop Protection and Rural Environment, University of Thessaly, at 25 °C, 65% relative humidity (r.h.) and continuous darkness. For all beetle species, adult beetles <1 month old were used in the tests. For *O. surinamensis*, *T. castaneum* and *S. oryzae*, the rearing media were oats, wheat flour and whole wheat kernels, respectively. For each of the above species, the standard laboratory populations (LB) were used, as well as field populations, namely for *O. surinamensis* Def, *T. castaneum* QTC931 and *S. oryzae* 3Tusc. All laboratory populations were collected from Greece and maintained in LEAZ for more than 20 years, while *O. surinamensis* Def, *T. castaneum* QTC931 and *S. oryzae* 3Tusc were collected from Spain, Australia and Italy, respectively, and reared in LEAZ since 2017.

### 2.2. Detection of Phosphine Resistance

The Food and Agriculture Organization (FAO) protocol, as described by the FAO Plant Protection Bulletin (FAO 1975), and modified by Agrafioti et al. [8], was used for the evaluation of the occurrence of phosphine resistance for each population. In brief, twenty adults of each of the tested species and populations were placed in a 1.5 L glass jar and exposed to phosphine concentration of 30 ppm for 20 h. After the termination of the exposure interval, active—i.e., capable of coordinated movement—and immobilized—i.e., not capable of coordinated movement—adults were recorded, as described by Athanassiou et al. [23]. The whole procedure was repeated three times (three replicates), with three sub-replicates each, with new phosphine production on each replicate, as suggested by Agrafioti et al. [8].

### 2.3. Nitrogen Treatment

**Figure 1:**
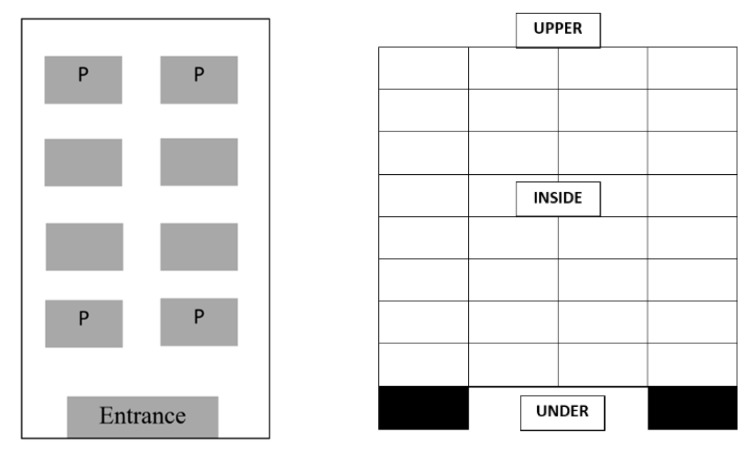
Distribution of the pallets (**left**) inside the chamber (with «P» are the pallets in which insect vials were placed) and locations in each pallet in which vials with insects were placed (upper, inside and under the pallet). Black color indicates the legs of the pallet (**right**).

The trials were conducted in a commercial facility (Agricultural Cooperatives’ Union of Aeghion S.A.) between May 2018 and October 2019. Nine trials were conducted inside nitrogen chambers (length 17 m, width 3.95 m, high 2.90 m) (AgroSpeCom Ltd., Thessaloniki, Greece), in which nitrogen was introduced through an incorporated nitrogen generator (Ali 4100). High purity nitrogen (99.0% N_2_ and 1.0% O_2_) was produced from air through a pressure swing adsorption (PSA) process and pumped inside the chambers at a maximum flow rate of 72 m^3^/h. Each chamber was filled with eight pallets carrying boxes of packed currants (*Vitis vinifera vinifera* L. var. Apyrena, each pallet contained 64 boxes of 12.5 kg each) (Figure 1) The dimension of each pallet was 1.60 in high, 0.80 in width and 1.20 in length. Based on a previous study [18], we selected different conditions for each trial (temperature, exposure time) which are presented in Table 1.

From each species and population (susceptible and resistant), insects were placed in plastic vials (3 cm in diameter, 8 cm in high, Rotilabo Sample tins Snap on lid, Carl Roth, Germany) for the trials (3 species × 2 populations × 3 replicates = 18 vials per location per pallet). The vials were perforated in the upper part. Two days before each trial, adults were taken from the cultures and ten individuals from each insect species and population were placed in the vials and were maintained in incubators at 25 °C, 65% r.h. Each vial was filled with 10 g of oat flakes for *O. surinamensis*, flour for *T. castaneum* and wheat kernels for *S. oryzae*. The series of vials were placed in four different pallets inside each chamber, with 4 different locations in each pallet—i.e., at the center of the pallets, at the upper part of the pallets and under the pallets (for distribution in each trial, see Figure 1, Table 1). For each species, there were three replicates in each location. A separate series of vials was placed outside of the chamber and used as controls.

After the termination of each trial, the chamber was opened, and all vials were transferred to the laboratory for assessment of adult mortality. All adults (dead and alive) were removed, and the vials were kept in incubators set at 25 °C, 65% r.h. and in continuous darkness. Observations on adult progeny were recorded after 65 days.

### 2.4. Data Analysis

All data, separately for each trial, insect species and population were subjected to an independent *t*-test, with insect mortality as the response variable. To determine the effect of location for each trial and insect species, data were subjected to a one-way ANOVA with insect mortality as the response variable and location as the main effect. Control mortality was generally low, so the data for control mortality were not used in the analysis. The same analysis approach was also followed in the case of progeny production counts. Means were separated by using the Student’s *t*-test at *p* < 0.05.

## 3. Results

### 3.1. Detection of Phosphine Resistance

Regarding the susceptible populations (LB) of all species, no active adults were found after the 20 h of exposure at 30 ppm. In contrast, for the field populations, survival was 100% after the termination of the exposure interval for *O. surinamensis* Def, *T. castaneum* QTC931 and *S. oryzae* 3Tusc.

### 3.2. Insect Mortality and Progeny Production

Control mortality of parental adults was negligible (<4% for all species). For all populations tested, complete control of the parental adults was achieved at all nitrogen trials for both laboratory and field populations of *O. surinamensis* and *S. oryzae* (results are not presented, 100% mortality), with the exception of *T. castaneum* LB and *T. castaneum* QTC931 in trials 3 and 5, respectively, which were carried out at either 28 °C and 3 d or 28 °C and 9 d (Table 2). No significant differences were noted among locations for the two populations of *T. castaneum* (Table 2).

Regarding progeny production in the control vials, this was 95.0 ± 38.7, 109.8 ± 17.6, 47.8 ± 12.5, 48.6 ± 6.2, 108.7 ± 17.9 and 143.7 ± 18.8 adults per vial for *O. surinamensis* LB, *O. surinamensis* Def, *T. castaneum* LB, *T. castaneum* QTC931, *S. oryzae* LB and *S. oryzae* 3Tusc, respectively. Progeny production was extremely low for *O. surinamensis* LB and progeny production was completely suppressed for *O. surinamensis* Def (Table 3). Progeny production was recorded for *T. castaneum* LB only in one location in trial 3 at 28 °C for 3 days. Moreover, for *T. castaneum* QTC931, the progeny production of which was recorded only in trial 6 at 28 °C for 3 d (Table 4). For some locations, progeny production of both populations of *S. oryzae* was high, but only in trial 2, which was performed at 28 °C for 3 d (Table 5). Moreover, significant differences were noted among locations only in the case of trial 2 and only for *S. oryzae* 3Tusc (Table 5).

## 4. Discussion

Considering the literature available, this is the first study that describes the insecticidal effect of nitrogen against phosphine susceptible and resistant populations. Our data clearly indicate that nitrogen treatment can be used successfully for the control of major stored product insects that are resistant to phosphine. Based on our results, resistant populations of *O. surinamensis*, *T. castaneum* and *S. oryzae* are highly susceptible to nitrogen, at the temperatures and exposure levels tested here. The current results stand in accordance with the data previously reported by Athanassiou et al. [18] for stored currants, indicating that complete control is achievable at intervals that are shorter than 7 days, provided that nitrogen is applied in combination with elevated temperatures. Moreover, in that study, the authors noted such an application did not affect the basic properties of the commodity, and, to some extent, nitrogen reduced mold density [18].

The overall progeny production was much lower at longer exposures, than at short ones. In the same way, progeny production was recorded at 28 °C, but not at 40 °C. Considering the significant effect of temperature in shortening the duration of the application, and taking into account that the commodity is not affected [18], shorter application times are feasible and can be further implemented in “real world” modified atmosphere applications. The “traditional” protocols of the application of nitrogen that have been proposed during recent decades suggest exposure intervals that are 7 days or longer [24]. In addition to this interval, the operator must consider some additional days that are required to reach the target of 1% oxygen, which makes the overall duration of the application at least 10 days, and in many cases much longer. This constitutes nitrogen a long-lasting application, that requires application times that are much longer than those of the typical methods that are used for the disinfestations of durable commodities, such as phosphine [25]. The increase in temperatures is not feasible in the application of nitrogen in large facilities, such as silos [19], but can be easily controlled in chambers [18]. Our experiments show that temperature is maybe equally important as exposure. Elevated temperatures drastically increase insect stress, even if applied alone, affecting different mechanisms, such as water loss and membrane disorders [26,27]. In this context, even if applied alone, heat can effectively control insects in different types of storage and processing facilities [28,29,30].

Apart from testing parental individuals, progeny production capacity of the populations was tested. In many studies on aerial insecticides, the efficacy trials are focused on using parental adults, which may provide a false impression that the application was successful, given that for many species, adult is the most susceptible life stage [31,32]. In addition, laboratory handling to provide the individuals that are to be exposed is likely to cause an additional stress/damage, especially in the case of eggs, which may give eventually high mortality levels that do not correspond to “real world” applications. In an earlier study with a commercial fumigation with phosphine, Aulicky et al. [33] exposed adults of *T. confusum* that were left to oviposit in flour for some interval before the initiation of the application, and, despite the fact that the application was effective for the parental adults, immature emergence was recorded in the commodity. The same approach was used in the current trials, and we saw that, despite its high efficacy, the method did not totally suppress progeny production in all combinations tested. Moreover, the specific vials where progeny production was recorded were the same ones where parental mortality was complete, suggesting that even a short oviposition period prior to death can support a post-exposure infestation. From a practical point of view, the utilization in similar commercial trials of small product quantities that all life stages may coexist could be a more realistic approach than separating eggs and immatures before the initiation of the application [33].

We selected the level of 1% of oxygen, considering its efficacy, based on previous works [14,16,17,18]. In fact, oxygen levels that were lower than 1% may not necessarily result in faster mortality levels. Navarro [16] reported that complete mortality of *S. oryzae* was achieved faster at 1% than at 0.1 or 2% oxygen under the same conditions. Higher oxygen levels, however, are also lethal, but variations among life stages may be more diverse. Tunc and Navarro [34] noted that complete mortality of *T. confusum* eggs was achieved at 2–4% oxygen in contrast with adults which survived to 4% oxygen for 96 h. For the khapra beetle, *Trogoderma granarium* Everts (Coleoptera: Dermestidae), Vassilakos et al. [35] found that adults of this species were more susceptible to conditions of low oxygen than to elevated carbon dioxide.

The survival/progeny patterns noted here were not correlated with the phosphine resistance level of the populations tested, and hence, nitrogen was effective regardless of the population. In this regard, nitrogen, at the conditions evaluated here, can be used effectively in programs and strategies that deal with phosphine resistance mitigation measures. As controlled atmospheres can be applied directly on the commodity, nitrogen may be considered as a method that can be rotated with phosphine, or even used as a standalone treatment. At the same time, the use of this method at elevated temperatures (e.g., 40 °C), provided that there are no negative interactions with the commodity, can be effective for the control of resistant populations at intervals that are directly comparable with commercial phosphine applications.

## 5. Conclusions

The findings of the present study illustrate that low oxygen can be an effective alternative method against phosphine-susceptible and -resistant populations of *O. surinamensis*, *T. castaneum* and *S. oryzae*. Moreover, temperature and duration of application are critical for the effective control of these species. Progeny production was lower at longer exposures than at shorter ones. The conditions that were tested here can be used in pest management strategies in order to mitigate phosphine resistance. Finally, low oxygen could be a variable tool in pest management strategies for ensuring control of major stored product insects that are resistant to phosphine.

## Figures and Tables

**Table 1:** Distribution of the vials with insects in the pallets (at the center of the pallets, at the upper part of the pallets and under the pallets), along with the conditions for each trial. Locations were slightly varied from trial to trial based on accessibility criteria.

Trial Number	Temperature/Exposure	Location 1	Location 2	Location 3	Location 4
Trial 1	28 °C, 3 d	Inside	Under	Upper	Inside
Trial 2	28 °C, 3 d	Inside	Under	Upper	Inside
Τrial 3	28 °C, 3 d	Inside	Under	Upper	Inside
Trial 4	28 °C, 9 d	Inside	Inside	Upper	Under
Trial 5	28 °C, 9 d	Inside	Inside	Upper	Under
Trial 6	28 °C, 9 d	Inside	Inside	Upper	Under
Trial 7	40 °C, 2.5 d	Inside	Inside	Upper	Under
Trial 8	40 °C, 2.5 d	Inside	Inside	Upper	Under
Trial 9	40 °C, 2.5 d	Inside	Inside	Upper	Under

**Table 2:** Mean mortality (± SE) of two populations of *T. castaneum* (LB, QTC931), with different susceptibility to phosphine, exposed to nitrogen in nine different trials (for conditions for each trial, see Table 1). For each trial, vials with insects were placed in four different locations (L1–L4, see Table 1).

Trial Number	*T. castaneum* LB				*T. castaneum* QTC931			
	L1	L2	L3	L4	L1	L2	L3	L4
Trial 1	100.0 ± 0.0	100.0 ± 0.0	100.0 ± 0.0	100.0 ± 0.0	100.0 ± 0.0	100.0 ± 0.0	100.0 ± 0.0	100.0 ± 0.0
Trial 2	100.0 ± 0.0	100.0 ± 0.0	100.0 ± 0.0	100.0 ± 0.0	100.0 ± 0.0	100.0 ± 0.0	100.0 ± 0.0	100.0 ± 0.0
Trial 3	96.7 ± 3.3	96.7 ± 3.3	100.0 ± 0.0	100.0 ± 0.0	100.0 ± 0.0	100.0 ± 0.0	100.0 ± 0.0	100.0 ± 0.0
Trial 4	100.0 ± 0.0	100.0 ± 0.0	100.0 ± 0.0	100.0 ± 0.0	100.0 ± 0.0	100.0 ± 0.0	100.0 ± 0.0	100.0 ± 0.0
Trial 5	100.0 ± 0.0	100.0 ± 0.0	100.0 ± 0.0	100.0 ± 0.0	100.0 ± 0.0	96.7 ± 3.3	100.0 ± 0.0	100.0 ± 0.0
Trial 6	100.0 ± 0.0	100.0 ± 0.0	100.0 ± 0.0	100.0 ± 0.0	100.0 ± 0.0	100.0 ± 0.0	100.0 ± 0.0	100.0 ± 0.0
Trial 7	100.0 ± 0.0	100.0 ± 0.0	100.0 ± 0.0	100.0 ± 0.0	100.0 ± 0.0	100.0 ± 0.0	100.0 ± 0.0	100.0 ± 0.0
Trial 8	100.0 ± 0.0	100.0 ± 0.0	100.0 ± 0.0	100.0 ± 0.0	100.0 ± 0.0	100.0 ± 0.0	100.0 ± 0.0	100.0 ± 0.0
Trial 9	100.0 ± 0.0	100.0 ± 0.0	100.0 ± 0.0	100.0 ± 0.0	100.0 ± 0.0	100.0 ± 0.0	100.0 ± 0.0	100.0 ± 0.0

No significant differences were noted between the two populations within each trial (*t*-test at 0.05). No significant differences were noted among locations in each trial (HSD test at 0.05).

**Table 3:** Progeny production (mean number of adults ± SE/vial) in vials containing parental adults of two populations of *O. surinamensis* (LB, Def), with different susceptibility to phosphine, exposed to nitrogen in nine different trials (for conditions for each trial, see Table 1). For each trial, vials with insects were placed in four different locations (L1–L4, see Table 1).

Trial Number	*O. surinamensis* LB				*O. surinamensis* Def			
	L1	L2	L3	L4	L1	L2	L3	L4
Trial 1	0.0 ± 0.0	0.3 ± 0.3	0.7 ± 0.7	0.0 ± 0.0	0.0 ± 0.0	0.0 ± 0.0	0.0 ± 0.0	0.0 ± 0.0
Trial 2	0.0 ± 0.0	0.0 ± 0.0	0.0 ± 0.0	0.0 ± 0.0	0.0 ± 0.0	0.0 ± 0.0	0.0 ± 0.0	0.0 ± 0.0
Trial 3	0.0 ± 0.0	0.0 ± 0.0	0.0 ± 0.0	0.0 ± 0.0	0.0 ± 0.0	0.0 ± 0.0	0.0 ± 0.0	0.0 ± 0.0
Trial 4	0.0 ± 0.0	0.0 ± 0.0	0.0 ± 0.0	0.0 ± 0.0	0.0 ± 0.0	0.0 ± 0.0	0.0 ± 0.0	0.0 ± 0.0
Trial 5	0.0 ± 0.0	0.0 ± 0.0	0.0 ± 0.0	0.0 ± 0.0	0.0 ± 0.0	0.0 ± 0.0	0.0 ± 0.0	0.0 ± 0.0
Trial 6	0.0 ± 0.0	0.0 ± 0.0	0.0 ± 0.0	0.0 ± 0.0	0.0 ± 0.0	0.0 ± 0.0	0.0 ± 0.0	0.0 ± 0.0
Trial 7	0.3 ± 0.3	0.0 ± 0.0	0.0 ± 0.0	0.0 ± 0.0	0.0 ± 0.0	0.0 ± 0.0	0.0 ± 0.0	0.0 ± 0.0
Trial 8	0.0 ± 0.0	0.0 ± 0.0	0.0 ± 0.0	0.0 ± 0.0	0.7 ± 0.7	0.0 ± 0.0	0.0 ± 0.0	0.0 ± 0.0
Trial 9	0.0 ± 0.0	0.0 ± 0.0	0.0 ± 0.0	0.0 ± 0.0	0.0 ± 0.0	0.0 ± 0.0	0.0 ± 0.0	0.0 ± 0.0

No significant differences were noted between the two populations within each trial (*t*-test at 0.05). No significant differences were noted among locations in each trial (HSD test at 0.05).

**Table 4:** Progeny production (mean number of adults ± SE/vial) in vials containing parental adults of two populations of *T. castaneum* (LB, QTC931), with different susceptibility to phosphine, exposed to nitrogen in nine different trials (for conditions for each trial, see Table 1). For each trial, vials with insects were placed in four different locations (L1–L4, see Table 1).

Trial Number	*T. castaneum* LB				*T. castaneum* QTC931			
	L1	L2	L3	L4	L1	L2	L3	L4
Trial 1	0.0 ± 0.0	0.0 ± 0.0	0.0 ± 0.0	0.0 ± 0.0	0.0 ± 0.0	0.0 ± 0.0	0.0 ± 0.0	0.0 ± 0.0
Trial 2	0.0 ± 0.0	0.0 ± 0.0	0.0 ± 0.0	0.0 ± 0.0	0.0 ± 0.0	0.0 ± 0.0	0.0 ± 0.0	0.0 ± 0.0
Trial 3	20.3 ± 20.3	0.0 ± 0.0	0.0 ± 0.0	0.0 ± 0.0	0.0 ± 0.0	0.0 ± 0.0	0.0 ± 0.0	0.0 ± 0.0
Trial 4	0.0 ± 0.0	0.0 ± 0.0	0.0 ± 0.0	0.0 ± 0.0	0.0 ± 0.0	0.0 ± 0.0	0.0 ± 0.0	0.0 ± 0.0
Trial 5	0.0 ± 0.0	0.0 ± 0.0	0.0 ± 0.0	0.0 ± 0.0	0.0 ± 0.0	0.0 ± 0.0	0.0 ± 0.0	0.0 ± 0.0
Trial 6	0.0 ± 0.0	0.0 ± 0.0	0.0 ± 0.0	0.0 ± 0.0	0.0 ± 0.0	4.0 ± 4.0	0.0 ± 0.0	0.0 ± 0.0
Trial 7	0.0 ± 0.0	0.0 ± 0.0	0.0 ± 0.0	0.0 ± 0.0	0.0 ± 0.0	0.0 ± 0.0	0.0 ± 0.0	0.0 ± 0.0
Trial 8	0.0 ± 0.0	0.0 ± 0.0	0.0 ± 0.0	0.0 ± 0.0	0.0 ± 0.0	0.0 ± 0.0	0.0 ± 0.0	0.0 ± 0.0
Trial 9	0.0 ± 0.0	0.0 ± 0.0	0.0 ± 0.0	0.0 ± 0.0	0.0 ± 0.0	0.0 ± 0.0	0.0 ± 0.0	0.0 ± 0.0

No significant differences were noted between the two populations within each trial (*t*-test at 0.05). No significant differences were noted among locations in each trial (HSD test at 0.05).

**Table 5:** Progeny production (mean number of adults ± SE/vial) in vials containing parental adults of two populations of *S. oryzae* (LB, 3Tusc), with different susceptibility to phosphine, exposed to nitrogen in nine different trials (for conditions for each trial, see Table 1). For each trial, vials with insects were placed in four different locations (L1–L4, see Table 1).

Trial Number	*S. oryzae* LB				*S. oryzae* 3Tusc			
	L1	L2	L3	L4	L1	L2	L3	L4
Trial 1	0.0 ± 0.0	0.3 ± 0.3	0.3 ± 0.3	0.0 ± 0.0	0.0 ± 0.0	0.0 ± 0.0	0.3 ± 0.3	0.0 ± 0.0
Trial 2	94.0 ± 43.6A	0.0 ± 0.0B	0.0 ± 0.0B	0.0 ± 0.0B	162.0 ± 39.1A	0.0 ± 0.0B	0.0 ± 0.0B	0.0 ± 0.0B
Trial 3	0.0 ± 0.0	0.3 ± 0.3	0.3 ± 0.3	0.0 ± 0.0	1.0 ± 0.6	0.0 ± 0.0	0.3 ± 0.3	1.0 ± 0.6
Trial 4	0.0 ± 0.0	0.0 ± 0.0	0.0 ± 0.0	0.0 ± 0.0	0.0 ± 0.0	0.0 ± 0.0	0.0 ± 0.0	0.0 ± 0.0
Trial 5	0.0 ± 0.0	0.0 ± 0.0	0.0 ± 0.0	0.0 ± 0.0	0.0 ± 0.0	0.0 ± 0.0	0.0 ± 0.0	0.0 ± 0.0
Trial 6	0.0 ± 0.0	0.0 ± 0.0	0.0 ± 0.0	0.0 ± 0.0	0.0 ± 0.0	0.0 ± 0.0	0.0 ± 0.0	0.0 ± 0.0
Trial 7	0.0 ± 0.0	0.0 ± 0.0	0.0 ± 0.0	0.0 ± 0.0	0.0 ± 0.0	0.0 ± 0.0	0.0 ± 0.0	0.0 ± 0.0
Trial 8	0.0 ± 0.0	0.0 ± 0.0	0.0 ± 0.0	0.0 ± 0.0	0.0 ± 0.0	0.0 ± 0.0	0.0 ± 0.0	0.0 ± 0.0
Trial 9	0.0 ± 0.0	0.0 ± 0.0	0.0 ± 0.0	0.0 ± 0.0	0.0 ± 0.0	0.0 ± 0.0	0.0 ± 0.0	0.0 ± 0.0

Where no letters exist, no significant differences were noted between the two populations within each trial (*t*-test at 0.05). Μeans followed by the same uppercase do not differ significantly among locations in each trial and population; where no letters exist, no significant differences were noted (HSD test at 0.05).
